# Prediction model for unplanned extubation of thoracoabdominal drainage tube in postoperative inpatients: a retrospective study

**DOI:** 10.1186/s40001-025-02748-4

**Published:** 2025-06-09

**Authors:** Yushu Sun, Xiuping Li, Jia Xu, Xiaojie Zhang, Fanglei Gu, Hongying Pan

**Affiliations:** Nursing Department, Sir Run Run Shaw Hospital & Shanghai Artificial Intelligence Laboratory, Zhejiang University School of Medicine, Qingchun Road, Shangcheng District, Hangzhou, Zhejiang Province China

**Keywords:** Unplanned extubation, Prediction model, Thoracoabdominal drainage tube, Risk factors

## Abstract

**Background:**

It is crucial to identify the risk factors for unplanned extubation (UEX) in thoracoabdominal drainage tubes as early as possible and establish applicable risk prediction model to reduce the incidence of UEX.

**Methods:**

A retrospective survey of patients who underwent Thoracoabdominal drainage tubes placement at a tertiary hospital was conducted in Zhejiang Province, China, between January 2020 and January 2023. A training set was established to build the predictive model and conduct internal validation, which was assessed for discrimination using ROC curves and for Calibration using the Hosmer–Lemeshow test and Calibration curves. A nomogram was constructed to visually present the results of the logistic regression analysis. An external validation dataset was created for assessing the external validation of the model.

**Results:**

a total of 2220 patients were enrolled. Multiple logistic regression analysis showed that negative pressure ball drainage, adhesive fixation method, self-care ability (self-care vs. complete dependence), self-care ability (partial dependence vs. complete dependence), and Thoracoabdominal drainage tubes were statistically significant factors associated with UEX (*P* < 0.05).The predictive model equation was as follows: a = 0.95–1.66 × drainage method + 2.45 × fixation method −4.17 × self-care ability (self-care vs. complete dependence) −2.79 × self- care ability (partial dependence vs. complete dependence).In the internal validation, the AUC was 0.897 (95% CI = 0.87–0.92; *P* < 0.001), with a sensitivity of 0.75 and specificity of 0.93, indicating a high level of discrimination for the model. The Hosmer–Lemeshow test yielded a chi-square (χ^2^) value of 2.823 with 8 degrees of freedom and a *P*-value of 0.945, indicating high accuracy of the model. In the external validation, the AUC was 0.839 (95% CI = 0.75–0.93; *P* < 0.001), with a sensitivity of 0.73 and specificity of 0.96. The Hosmer–Lemeshow test yielded a χ^2^ value of 12.85 with 8 degrees of freedom and a *P*-value of 0.117. The DCA plot shows that the DCA curve is consistently higher than the two extreme curves, indicating a good fit of the model.

**Conclusion:**

The predictive model for the risk of unplanned extubation of thoracoabdominal drainage tubes in postoperative patients demonstrates good discrimination and Calibration. It can provide reference for clinical nursing staff in predicting the risk and early development of personalized preventive strategies for drainage tube UEX.

## Background

The use of indwelling drainage tubes is a common postoperative treatment method in surgery, aimed at promptly draining exudates from the wound or surgical site, promoting the restoration of the surgical area to a normal state, and facilitating the early detection of complications such as bleeding and infection in the surgical area [1, 2]. Therefore, the quality of tube care directly impacts the prognosis and outcome of patients. Unplanned extubation (UEX) refers to the premature removal of a tube due to accidental dislodgment or negligence before meeting the extubation criteria [3]. The incidence of UEX is reported to be 1.1 to 4.3% in foreign countries [4, 5] and 4.5 to 22.1% in China [6], with drainage tubes being one of the main types of tubes associated with UEX [6]. Notably, over 90% of UEX cases are attributed to self-removal by patients [7]. UEX may cause local tissue damage, bleeding, infection, increase patient suffering, prolong hospital stay, and raise medical expenses [8, 9]. Both the "Hospital Catheter Care Quality Evaluation Criteria" [10] and the "Patient Safety Goals (2019 Edition)" [11] explicitly point out that hospitals should establish a UEX risk prediction and assessment process. Currently, scholars domestically and abroad often investigate UEX as a whole to explore its risk factors and prevention strategies [12], but the characteristics of UEX for different types of tubes are heterogeneous. There is limited research on the risk of UEX for postoperative thoracoabdominal drainage tubes. A study analyzing the action characteristics of UEX in ICU patients found that upper limb movements accounted for 82.1% of the intention to extubate [13], and the thoracoabdominal drainage tubes are closest to the upper limbs, posing a very high risk of UEX. Therefore, it is crucial to identify the risk factors for UEX in thoracoabdominal drainage tubes as early as possible and establish a clinically applicable risk prediction model to reduce the incidence of UEX. Existing research on UEX related to thoracoabdominal drainage tubes mostly focuses on causal analysis, continuous quality improvement, and improvements in fixation methods, with no reports on risk prediction models. This paper integrates literature reports and common risk factors in clinical practice. By conducting a retrospective case–control study, it constructs a risk prediction model for unplanned extubation of thoracoabdominal drainage tubes after surgery, providing a basis for healthcare professionals to objectively assess the risks, establish a risk assessment system for tube routes, and develop intervention strategies.

### Study population and data collection

This study was conducted by selecting patients who underwent surgery and had thoracic or abdominal drainage tubes placed in a tertiary hospital's surgical ward in Zhejiang Province, China. Patients were consecutively included if they met criteria, ensuring representativeness without intentional selection bias. The inclusion criteria for study subjects were as follows: (1) hospitalized patients with thoracic or abdominal drainage tubes in place; (2) complete demographic and clinical data available; (3) age of 18 years or older. Exclusion criteria included: (1) a history of psychiatric illness; (2) hospitalization for less than 24 h; (3) incomplete information.

For the training set, which was used to establish the model and conduct internal validation, data were collected from patients admitted between January 2020 and January 2022. For the external validation set, data were collected from patients admitted between February and October 2023. This external validation set was used to validate the model's performance in a different timeframe.

### Study material

For patients meeting the inclusion and exclusion criteria, clinical data were retrospectively collected through the hospital's electronic medical record system. This included demographic data (gender, age, BMI, educational level, occupation, type of health insurance), medical history (previous surgery, previous tube removal), current hospitalization status (whether transferred from ICU), patient status (level of consciousness, presence of caregiver, physical restraints, use of sedatives, use of analgesics, self-care ability), and catheter-related information (site of catheter insertion, drainage method, fixation method, leakage around the catheter, skin condition around the catheter, frequency of catheter maintenance).

### Statistical analysis

A database was established using Excel for data entry and organization, and statistical analysis was conducted using R software (version 4.2.2). Continuous variables were presented as mean ± standard deviation (x̄ ± s) or median (P25, P75) and compared using independent samples t-test or non-parametric tests. Categorical data were described using frequencies and percentages and compared using chi-square tests or rank-sum tests. Factors showing statistical significance in univariate analysis were screened. Variables with *P* < 0.05 in univariate analysis were included in the multivariate analysis, where multivariate logistic regression analysis (stepwise forward method) was used to determine the final risk factors and construct a predictive model. The alpha value for entering the model was set at 0.05, and the alpha value for excluding variables was set at 0.10.

In internal validation, Bootstrap resampling was used, and the AUC was calculated to evaluate the model's discriminatory power. The Hosmer–Lemeshow test and Calibration curves were used to assess the model's Calibration. Variables with P < 0.05 in the regression analysis were included as independent predictors in the nomogram model to display the probability of UEX.

In external validation, the AUC was calculated using the ROC curve to evaluate the model's discriminatory power. The Hosmer–Lemeshow test and Calibration curves were used to assess the model's Calibration. The Decision Curve Analysis (DCA) was utilized to evaluate the practicality and benefits of the predictive model in clinical decision-making. All tests were two-sided, and the significance level was set at α = 0.05.

### Ethical considerations

This study strictly adhered to the ethical principles outlined in the Declaration of Helsinki. The study protocol was reviewed and approved by the hospital's ethics committee (Approval Number: Hospital Ethics Review 2023 Research No. 0570).

## Results

### Basic characteristics

The modeling dataset for this study included a total of 2220 patients, of whom 1425 (64.2%) were male and 795 (35.8%) were female. The age range was 16 to 93 years, with a mean age of 63.95 ± 12.73 years. The Body Mass Index (BMI) ranged from 16 to 47, with a mean of 23.36 ± 5.87. Regarding employment, 416 patients (18.7%) were employed, and 1804 (81.3%) were unemployed. In terms of education, 514 patients (23.1%) had a high school degree or higher, while 1706 (76.9%) had a junior high school degree or lower. Insurance coverage was present in 1855 patients (83.6%), while 365 patients (16.4%) were self-paying. A history of previous surgery was reported in 1512 patients (68.1%), and no history of previous surgery in 708 patients (31.9%). A history of previous tube removal was reported in 33 patients (1.5%), and no history of previous tube removal in 2187 patients (98.5%).Regarding drainage methods, 420 patients (18.9%) used drainage bags, and 1800 patients (81.1%) used negative pressure balls. For fixation methods, 2030 patients (91.4%) used sutures, and 190 patients (8.6%) used patches. Caregivers were present for 2197 patients (99.0%), and absent for 23 patients (1.0%). Patients were transferred from the ICU in 333 cases (15.0%), and not transferred from the ICU in 1887 cases (85.0%). Thoracic drainage tubes were placed in 373 patients (16.8%), abdominal drainage tubes in 1817 patients (81.9%), and both types of drainage tubes were placed in 30 patients (1.4%).

Out of 2220 patients, 210 (9.5%) experienced UEX, while 2010 (90.5%) did not experience UEX.

### Analysis of risk factors for unplanned extubation

The univariate analysis revealed statistically significant differences (*P* < 0.05) between the UEX group and the non-UEX group in terms of age, type of insurance, history of previous surgery, history of previous tube removal, transfer from the ICU, site of catheter insertion, drainage method, fixation method, presence of a caregiver, physical restraint, use of sedatives, use of analgesics, level of consciousness, self-care ability, leakage around the catheter, skin condition around the catheter, and frequency of catheter maintenance (Table 1).

**Table 1:** Univariate analysis of risk factors for unplanned extubation

Variables	UEX (*n* = 65)	Non-UEX (*n* = 260)	Statistics	*P*
Gender				
Male	139	1286		
Female	71	724	0.404	0.525
Age				
≤ 64	67	938		
> 64	143	1072	16.73	< 0.0001
BMI				
≤ 25	169	1530		
> 25	41	480	2	0.1563
Education				
≤ 9 years	164	1542		
> 9 years	46	468	0.2032	0.6522
Occupation				
Employed	31	385		
Unemployed	179	1625	2.41	0.1206
Medical insurance				
Yes	152	1703		
No	58	307	21.09	< 0.0001
Surgical history				
Yes	1	707		
No	209	1303	105.39	< 0.0001
Extubation history				
Yes	196	1991		
No	14	19	42.5	< 0.0001
Transferred from ICU				
Yes	143	1744		
No	67	266	51.99	< 0.0001
Drainage method				
Drainage bags	143	277		
Negative pressure ball	67	1733	365.66	< 0.0001
Fixation methods				
Suture	101	1929		
Adhesive dressing	109	81	556.85	< 0.0001
Caregivers				
Yes	22	1		
No	36	168	17.58	< 0.0001
Physical restraint				
Yes	197	1993		
No	13	17	40.74	< 0.0001
Sedative				
Yes	195	1990		
No	15	20	46.31	< 0.0001
Analgesic				
Yes	54	849		
No	156	1161	21.52	< 0.0001
Consciousness				
Drowsiness/altered consciousness	17	5		
Fully awake	193	2005	119.31	< 0.0001
Self-care ability	58	12		
Complete dependence	58	12		
Partial dependence	343	80		
Self-care	72	1655	540.88	< 0.0001
Leakage				
Yes	141	1852		
No	69	158	129.42	< 0.0001
Skin condition around the catheter				
No abnormalities	195	1976		
Erythema and swelling	15	34	26.18	< 0.0001
Frequency of catheter maintenance				
Maintenance per shift	207	1972		
Daily maintenance	2	0		
Maintenance as needed	1	38	21.31	< 0.0001

The variables that showed statistical significance in the univariate analysis were entered into a multivariate Logistic regression model. The results of the Logistic regression analysis indicated that negative pressure ball drainage, adhesive fixation method, self-care ability (self-care vs. complete dependence), and self-care ability (partial dependence vs. complete dependence) were significantly associated with UEX (*P* < 0.05) (Table 2).

**Table 2:** Multivariate logistic regression analysis of risk factors for UEX

Variables	*B*	*SE*	Wald *χ*^*2*^	*P*	*OR*	95%*CI*
Intercept	0.95	0.58	2.70	0.1006	–	–
Drainage method (negative pressure ball)	− 1.66	0.20	67.84	< 0.001	0.19	0.13~0.28
Fixation methods (adhesive dressing)	2.45	0.22	119.88	< 0.001	11.59	7.47~17.97
Self-care ability (self-care VS complete dependence)	− 4.17	0.39	114.07	< 0.001	0.02	0.01~0.03
Self-care ability (partial dependence VS complete dependence)	− 2.79	0.40	48.97	< 0.001	0.06	0.03~0.13

### Construction and internal validation of a predictive model for UEX of thoracoabdominal drainage tubes in postoperative surgical patients

Based on the results of the multivariate regression analysis, we derived the partial regression coefficients for each independent variable and constructed a predictive model for UEX of thoracoabdominal drainage tubes in postoperative surgical patients. The calculation formula is as follows: ea/(1 + ea) × 100%, where e is the base of the natural logarithm, *a* = 0.95 − 1.66 × Drainage Method + 2.45 × Fixation Method − 4.17 × Self-Care Ability (Self-care vs. Complete Dependence) − 2.79 × Self-Care Ability (Partial Dependence vs. Complete Dependence).

**Fig. 1:**
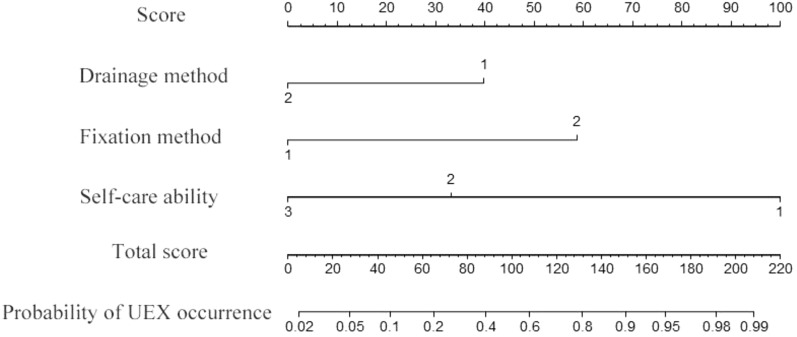
Nomogram predicting the probability of UEX

Using the R software, we plotted a nomogram to predict the probability of UEX (Fig. 1). In this model: Drainage Method: 1 = Drain Bag, 2 = Negative Pressure Ball; Fixation Method: 1 = Suture, 2 = Adhesive; Self-Care Ability: 1 = Complete Dependence, 2 = Partial Dependence, 3 = Self-care. In the nomogram, points corresponding to each category are summed up to obtain a total score, which can then be converted into the probability of unplanned extubation. The percentage value corresponding to the total score represents the probability of UEX in postoperative surgical patients with thoracoabdominal drainage tubes. For instance, a patient with "negative pressure ball drainage (Score: (Drainage Method = 2) = 0)", " Adhesive fixation (Score: (Fixation Method = 2) = 60)", "partial dependence self-care ability (Score: (Self-Care Ability = 2) = 35)", total score = 0 + 60 + 35 = 95. In Fig. 1, a total score of 95 corresponds to a probability of UEX occurrence of 0.45, indicating that this patient has a 45% probability of UEX.

**Fig. 2:**
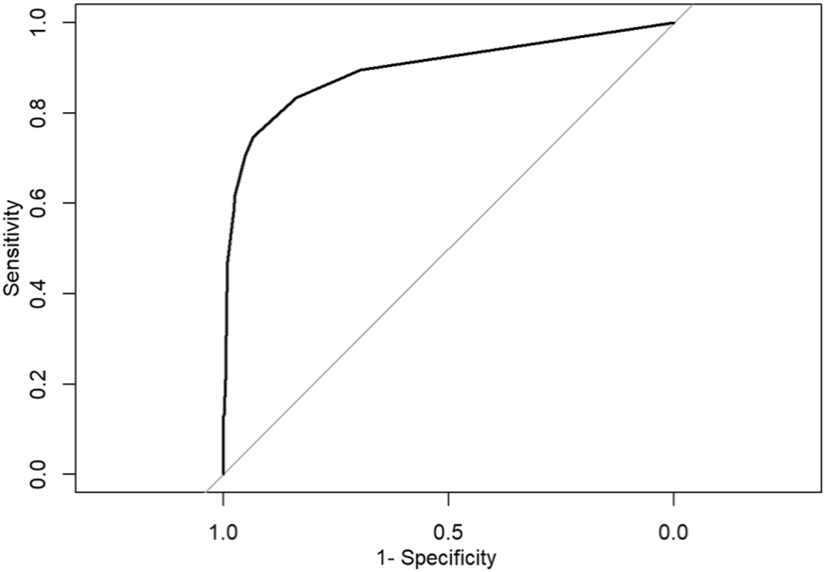
ROC curve for the predictive model

We also plotted the receiver operating characteristic (ROC) curve and calculated the area under the curve (AUC) to assess the discriminatory ability of the model. The AUC was 0.897 (95% CI = 0.87 to 0.92; *P* < 0.001), with a sensitivity of 0.75 and specificity of 0.93 (Fig. 2), indicating a high degree of discrimination.

**Fig. 3:**
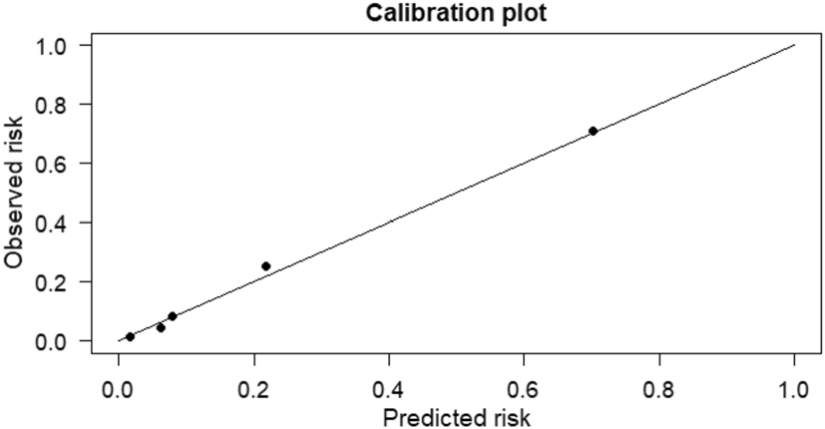
Calibration curve for the predictive model

To evaluate the Calibration of the model, we used a Calibration curve (Fig. 3). The Hosmer–Lemeshow test yielded a chi-squared value of 2.823 with 8 degrees of freedom, resulting in a *P*-value of 0.945, suggesting a high level of accuracy for the predictive model.

### External validation of the predictive model

A total of 560 patients who met the inclusion criteria were included in the study, of which 53 patients experienced unplanned extubation and 507 had planned extubation.

Comparisons were made between the training set and the external validation set regarding basic demographic information such as age, gender, educational level, occupation, and health insurance. No statistically significant differences were found, indicating comparability between the two groups (Table 3).

**Table 3:** Comparison of basic demographic information between training dataset and external validation dataset

Variables	Total (n = 2780)	External validation set (n = 560)	Training dataset (n = 2220)	Statistics	*P*
Age, mean ± standard deviation	63.99 ± 12.81	63.35 ± 13.57	64.15 ± 12.60	*t* = − 1.26	0.21
Gender					
Male	988 (35.54)	193 (34.46)	795(35.84)		
Female	1792 (64.46)	367 (65.54)	1425 (64.16)	χ^2^ = 0.37	0.54
Education					
≤ 9 years	2139 (76.94)	433 (77.32)	1706 (76.86)		
> 9 years	641 (23.06)	127 (22.68)	514 (23.14)	χ^2^ = 0.05	0.82
Occupation					
Employed	530 (19.06)	114 (20.36)	416 (18.73)		
Unemployed	2250 (80.94)	446 (79.64)	1804 (81.27)	χ^2^ = 0.77	0.38
Medical insurance					
Yes	2310 (83.09)	455 (81.25)	1855 (83.57)		
No	470 (16.91)	105 (18.75)	365 (16.43)	χ^2^ = 1.71	0.19

**Fig. 4:**
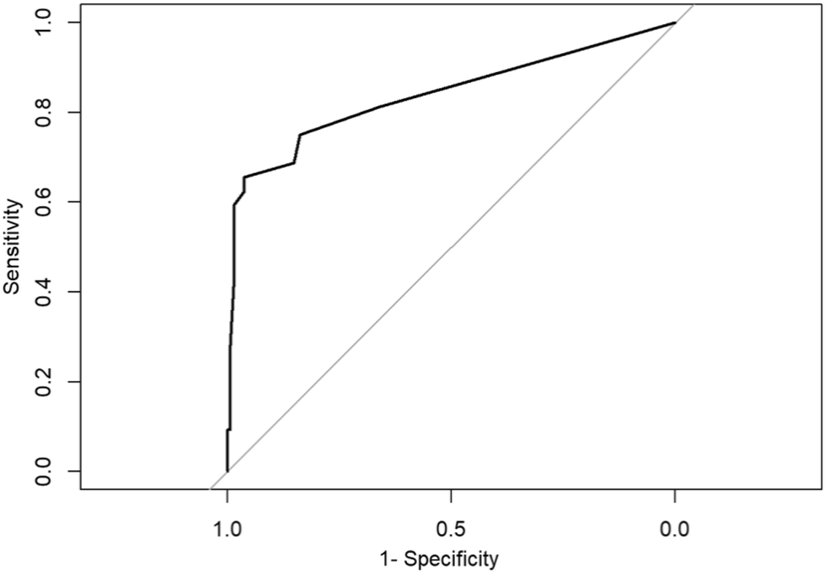
ROC curve for the predictive model of UEX

The ROC curve was plotted to assess the discriminatory ability of the predictive model for UEX, and the AUC was calculated. The AUC was 0.839 (95% CI = 0.75 to 0.93; *P* < 0.001), with a sensitivity of 0.73 and specificity of 0.96. These findings from the external validation demonstrate a high degree of discriminatory ability for the model (Fig. 4).

**Fig. 5:**
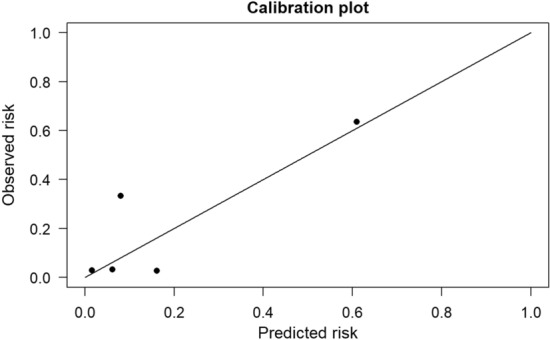
Calibration curve for the predictive model of UEX

The Calibration curve was also plotted to evaluate the Calibration of the model, i.e., the consistency between the predicted probabilities and the observed event rates. The Hosmer–Lemeshow goodness-of-fit test yielded a chi-squared value of 12.85 with 8 degrees of freedom (*P* = 0.1170), indicating good Calibration of the predictive model (Fig. 5).

**Fig. 6:**
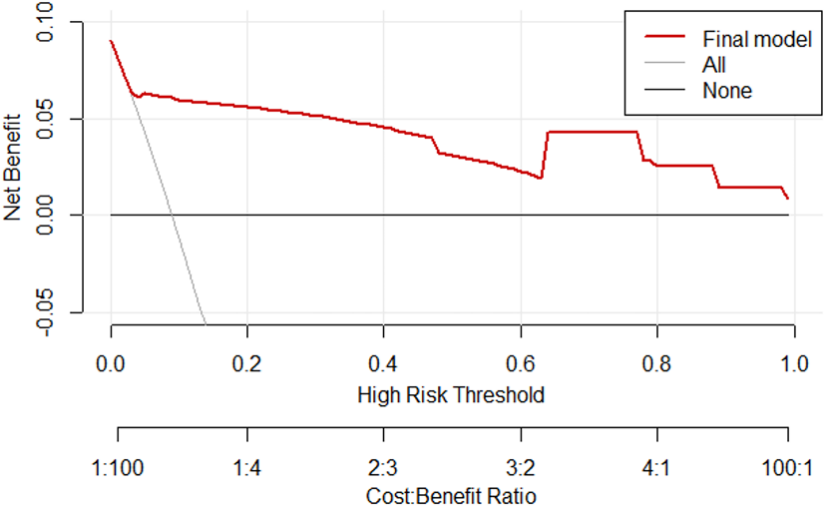
DCA for the predictive model of UEX

A DCA curve was plotted to evaluate the clinical utility of the model. As shown in Fig. 6, the red line represents the DCA curve of the predictive model developed in this study, while the light gray line labeled "All" represents the assumption that all subjects would experience UEX, and the thick black line labeled "None" represents the scenario where no patients would face the risk of extubation. The DCA curve is consistently higher than both extreme curves, suggesting that the model has a favorable fit and clinical utility.

## Discussion

### Good prediction performance of the risk prediction model for UEX

In this study, logistic regression analysis yielded a probability calculation formula. Based on these three risk factors, a nomogram for predicting the risk of UEX of thoracoabdominal drainage tubes in postoperative surgical patients was constructed, and internal and external validation of the model were performed. An AUC greater than 0.7 typically indicates good predictive ability, and the closer the AUC value is to 1, the better the model’s predictive accuracy [14]. The Hosmer–Lemeshow Chi-square test is used to assess the fit between the predicted probabilities generated by the model and the actual event probabilities. If* P* < 0.05, it indicates a significant difference between the predicted and actual values, suggesting poor predictive ability of the model. If *P* > 0.05, it indicates good predictive ability and reflects the real situation well [15].

The internal validation of this study showed a corrected AUC of 0.897, with a 95% confidence interval of 0.87–0.92, *P* < 0.001, sensitivity of 0.75, and specificity of 0.93, indicating a high degree of discrimination. The Hosmer–Lemeshow test resulted in *P* = 0.945, indicating high accuracy of the prediction model. Additionally, external validation was conducted using data from 560 patients collected during different time periods to investigate the model’s extrapolation and predictive abilities. The external validation results showed an AUC of 0.839, with a 95% confidence interval of 0.75–0.93, *P* < 0.001, and the Hosmer–Lemeshow test resulted in *P* = 0.117. Both validation Calibrations were close to the ideal curve. Therefore, the model consistently demonstrated good discriminative ability and predictive value.

Compared with internal validation, the area under the ROC curve in the external validation was slightly lower, mainly due to the use of modeling group data for internal validation, which often yields better predictive performance than external validation. The model had a sensitivity of 0.73 and a specificity of 0.96 when tested with validation data. This suggests that the model not only has good predictive ability but can also be applied to predict the risk of UEX in thoracoabdominal drainage tubes in postoperative surgical patients at other hospitals. The risk prediction model constructed in this study provides scientific reference for early identification of high-risk groups for UEX, facilitating timely targeted interventions to reduce the occurrence of UEX.

### Comparison of this model with domestic and international models for unplanned extubation warning and prediction

Currently, research on warning and prediction models for UEX is relatively limited. Previous studies have primarily focused on establishing risk assessment systems or tools to evaluate the risk of UEX [16–18]. In China, Wang Chao-nan et al. [18] constructed a risk assessment system for UEX using the Delphi method, including 12 first-level indicators and 33 s-level indicators to assess the risk of UEX in patients. Huang Su-su et al. [19] also employed the Delphi method to develop a risk assessment system for UEX, which included 5 first-level indicators, 13 s-level indicators, and 51 third-level indicators to quantitatively assess the risk of UEX in patients. Liu Qi-lan et al. [20] similarly used the Delphi method to form an unplanned extubation risk assessment scale for ICU patients and optimized its use through the ROC curve to determine the best cut-off point for identifying high-risk ICU patients for unplanned extubation. However, these risk assessment systems and scales involve many indicators and may lack practicality in clinical application, and most have not undergone reliability and validity testing, requiring further investigation. This study established a predictive warning model and utilized a nomogram to visualize the predictive model [21], thereby providing better individualized predictions and risk assessments in an intuitive, convenient, and visual manner.

In the few studies that have developed prediction models, a retrospective cohort study in Seoul, South Korea, investigated 6914 patients with catheters in the ICU and compared various machine learning algorithms such as random forest, logistic regression, artificial neural networks, and support vector machines using ROC curves. The results showed that the random forest model had the best fit, with an area under the ROC curve of 0.79, sensitivity of 0.95, and specificity of 0.39 [22]. Another study in South Korea investigated 5412 patients with tracheal tubes, of whom 60 experienced unplanned extubation. Logistic regression was used to construct three prediction models with different variables, with areas under the ROC curves ranging from 0.82 to 0.90, with the highest sensitivity of 0.56 and the highest specificity of 0.94 [23]. Compared to South Korean studies using machine learning, our model demonstrates comparable discriminative ability with simpler, clinically actionable variables. Unlike complex Delphi-derived scales, our nomogram integrates fewer indicators, enhancing bedside usability. This parsimony makes it suitable for resource-constrained settings, though multi-center validation is warranted. Our findings can provide theoretical basis for developing personalized intervention plans and risk prevention measures [22, 24–26]. Clinically, nursing staff can establish dynamic assessment schemes of varying intensity based on different risk levels of patients [27], thereby improving the efficiency and effectiveness of UEX prevention [23, 28]. The prediction model developed in this study incorporates easily obtainable predictive indicators and is simple to apply clinically, making it widely applicable.

### Characterization of risk factors for unplanned extubation of thoracoabdominal drainage tubes in postoperative surgical patients

#### Poor self-care ability is associated with high risk of UEX

Our study results show that, compared with completely dependent patients, patients with self-care ability and partially dependent patients have a lower incidence of UEX. Previous studies have indicated that elderly patients with functional impairments in daily living activities are the primary population experiencing unplanned extubation [29–31]. Patients without self-care ability or with poor self-care ability are typically older and have more severe conditions, and prolonged treatment and repeated manipulation of catheters increase the risk of unplanned extubation. Additionally, many elderly patients with poor self-care ability tend to be stubborn and emotionally unstable. As they age, their self-care ability, attention, and memory decline, making it difficult for them to fully understand health education provided by healthcare professionals [32, 33]. Furthermore, diseases and indwelling catheters inflict physical and psychological trauma on elderly patients, increasing the likelihood of self-extubation [34, 35]. This finding suggests the need to enhance the level of care for patients with poor self-care ability and increase the frequency of care [36], as well as strengthen health education for patient caregivers, to reduce the incidence of UEX.

#### High risk of UEX in patients using drain bags for drainage

Our study shows that the characteristics of the drainage tube influence the risk of UEX. Compared to drain bags, negative pressure ball drainage has a lower risk of UEX, possibly due to several reasons. First, chest and abdominal drainage tubes connected to drain bags are typically used when there is a large amount of abdominal drainage over a long period, such as with T-tube placement. Long-term use increases the likelihood of unplanned extubation. Second, the lubricating effect of drainage fluid, combined with increased leakage, erosion of sutures, and more frequent dressing changes, can lead to loose sutures, broken sutures, and dislodged dressings, increasing the risk of unplanned extubation. Third, negative pressure ball drainage is more convenient for patients to handle when moving or turning in bed, and when getting out of bed, the drainage ball is often placed in a pocket, making it easier to carry compared to a drain bag. However, because the drain bag relies on gravity for drainage, it must be positioned below the level of the catheter insertion site, and this positioning makes it difficult to access the bag when the patient turns. Despite this, the longer total length of the drainage tube and drain bag allows patients to move freely in bed without having to adjust the position of the bag. Nevertheless, changing body positions still increases the risk of compression and pulling on the tube. Studies ^37^have shown that drain bags, with their larger volume and longer connecting tubes, are less easy to reposition during patient movement, leading to a higher risk of accidental extubation, especially when the volume of drainage fluid is excessive. This finding underscores the need for enhanced health education and increased focus on routine catheter care for patients using drain bags.

#### High risk of UEX in patients using only dressings for fixation

The study results also indicate that using only dressings for fixation is associated with a high risk of UEX, which differs from some domestic research findings. Liu Qian [37] conducted an experimental study on neonates with nasogastric tubes, in which the experimental group received bundled care combined with 3 M transparent dressings, while the control group received standard care. The results showed a significantly lower rate of unplanned extubation in the experimental group. Our study results differ from these, possibly due to differences in study populations and methodologies. For chest and abdominal drainage tubes placed after surgery, suture fixation is performed during the operation, and clinical doctors receive training on proper suture fixation techniques, including requirements such as crossing knots, tying three or more knots, cutting sutures 0.5 cm above the knot, and ensuring adequate skin coverage [38]. Nurses also apply dressings for secondary fixation, using various types of dressings such as I-shaped dressings, claw-shaped dressings, and foam dressings, following the principle of high platform fixation. However, in cases of accidental extubation, it is often found that breakage of sutures is more common than ineffective dressing fixation, suggesting that suture fixation plays a crucial role in preventing unplanned extubation. Nurses should promptly intervene when they observe loosening of sutures due to prolonged catheter placement, fluid erosion, or suboptimal suture technique. For patients who are expected to have long-term catheter placement, are critically ill, or have experienced unplanned extubation, nurses should consider using suture fixation. The study results suggest that for patients with only dressing fixation, the frequency of catheter inspection should be increased, and timely maintenance and education should be provided to raise awareness about preventing extubation. Nursing staff should identify related risk factors early using the risk prediction model and implement improved fixation methods [39, 40], increase the frequency of care [9, 36], and observe and address issues promptly [41] to achieve high-quality catheter care.

### Limitations and implications

This study has certain limitations. First, the data originate from a single institution, limiting the generalizability of the results. Multi-center studies with larger sample sizes would help improve the model's predictive performance. Second, previous research has shown that health education and delirium can also affect unplanned extubation. Due to the lack of indicators reflecting effective health education and the absence of delirium scoring in hospitalized patients, these variables could not be objectively surveyed and extracted in this study, potentially affecting the model's predictive performance.

For clinical nursing, the predictive indicators in this study are easily accessible and do not require additional medical resource consumption. After further refinement of the model, the model can be embedded into electronic medical records as a real-time risk calculator, flagging high-risk patients during admission. Nurses can use the nomogram to prioritize care and tailor interventions. It is recommended to further investigate risk stratification schemes and develop tiered preventive measures based on different risk levels of patients, such as using negative pressure ball drainage, improving fixation methods, and enhancing the level of care for patients with poor self-care ability, to effectively prevent UEX of thoracoabdominal drainage tubes.

## Data Availability

According to Chinese law, the data are not publicly available, but are available on reasonable requests from the corresponding author.
